# Methods for health workforce projection model: systematic review and recommended good practice reporting guideline

**DOI:** 10.1186/s12960-024-00895-z

**Published:** 2024-04-17

**Authors:** John Tayu Lee, Ian Crettenden, My Tran, Daniel Miller, Mark Cormack, Megan Cahill, Jinhu Li, Tomoko Sugiura, Fan Xiang

**Affiliations:** 1 Institute of Health Policy and Management, College of Public Health, National Taiwan University, Taipei, Taiwan; 2 National Centre for Health Workforce Studies, College of Health and Medicine, Australian National University, Canberra, Australia; 3 Health Data Analytics Team, College of Health and Medicine, Australian National University, Canberra, Australia; 4 National Centre for Epidemiology and Population Health, Australian National University, Canberra, Australia

**Keywords:** Health workforce planning, Human resources for health, Forecasting model, Projection, Workforce modelling

## Abstract

**Background:**

Health workforce projection models are integral components of a robust healthcare system. This research aims to review recent advancements in methodology and approaches for health workforce projection models and proposes a set of good practice reporting guidelines.

**Methods:**

We conducted a systematic review by searching medical and social science databases, including PubMed, EMBASE, Scopus, and EconLit, covering the period from 2010 to 2023. The inclusion criteria encompassed studies projecting the demand for and supply of the health workforce. PROSPERO registration: CRD 42023407858.

**Results:**

Our review identified 40 relevant studies, including 39 single countries analysis (in Australia, Canada, Germany, Ghana, Guinea, Ireland, Jamaica, Japan, Kazakhstan, Korea, Lesotho, Malawi, New Zealand, Portugal, Saudi Arabia, Serbia, Singapore, Spain, Thailand, UK, United States), and one multiple country analysis (in 32 OECD countries). Recent studies have increasingly embraced a complex systems approach in health workforce modelling, incorporating demand, supply, and demand–supply gap analyses. The review identified at least eight distinct types of health workforce projection models commonly used in recent literature: population-to-provider ratio models (*n* = 7), utilization models (*n* = 10), needs-based models (*n* = 25), skill-mixed models (*n* = 5), stock-and-flow models (*n* = 40), agent-based simulation models (*n* = 3), system dynamic models (*n* = 7), and budgetary models (*n* = 5). Each model has unique assumptions, strengths, and limitations, with practitioners often combining these models. Furthermore, we found seven statistical approaches used in health workforce projection models: arithmetic calculation, optimization, time-series analysis, econometrics regression modelling, microsimulation, cohort-based simulation, and feedback causal loop analysis. Workforce projection often relies on imperfect data with limited granularity at the local level. Existing studies lack standardization in reporting their methods. In response, we propose a good practice reporting guideline for health workforce projection models designed to accommodate various model types, emerging methodologies, and increased utilization of advanced statistical techniques to address uncertainties and data requirements.

**Conclusions:**

This study underscores the significance of dynamic, multi-professional, team-based, refined demand, supply, and budget impact analyses supported by robust health workforce data intelligence. The suggested best-practice reporting guidelines aim to assist researchers who publish health workforce studies in peer-reviewed journals. Nevertheless, it is expected that these reporting standards will prove valuable for analysts when designing their own analysis, encouraging a more comprehensive and transparent approach to health workforce projection modelling.

**Supplementary Information:**

The online version contains supplementary material available at 10.1186/s12960-024-00895-z.

## Introduction

Effective health workforce planning is a key instrument for a resilient and sustainable health system that achieves its key objectives, including enhancing access, health outcomes, responsiveness, and reducing disparities [1]. Health workforce projection model is an integral element of health workforce planning, a crucial quantitative tool to attain the so-called ‘6 rights’ in the health workforce, e.g. ensuring the right number of professionals with the right skills, delivering the right services at the right time and place, all with right financial resources to address health workforce gap [2, 3]. Inaccurate health workforce projection and forecasting can result in workforce shortages or surpluses, leading to inadequate care, increased costs, and restricted access [1].

It is important to recognize that, in many health systems, health workforce challenges are not simply about the total number of healthcare professionals, but also a distributional one [4, 5]. In high-income countries, despite universal health coverage, it is not uncommon for the health system to suffer from severe shortages of healthcare professionals in specific specialties or regions [6, 7]. In low- and middle-income countries, where resource constraints often present significant challenges, achieving effective health workforce planning is further complicated by limited financial resources and the need to balance healthcare delivery with workforce sustainability, highlighted by the Sustainable Development Goals Target 3c [8, 9].

The analysis of the health workforce, though fundamentally a labour market study, exhibits attributes that distinguish it from other sectors. Notably, one of the significant challenges lies in the lengthy training period for health professionals, which can exceed a decade. The regulatory framework governing the health workforce introduces complexities, constraining its adaptability and response to short-term provider needs [10, 11]. The health workforce, itself operating as a complex system, is linked with many other sectors or systems in society, such as education, immigration, and public finance. Hence, achieving labour market equilibrium through the ‘invisible hand’ of demand and supply can be challenging, leading to both acute and chronic shortages or surpluses in the health workforce.

Over the past decade, the methodologies and techniques of health workforce projection and forecasting models have evolved more complex, particularly in methods aligning health workforce projection with underlying population health needs and health workforce requirements of various health service delivery models [12, 13]. However, there is a notable gap in the systematic review summarizing the development of these methodologies. While there may be numerous studies on health workforce projection and forecasting, there is a lack of systematic guidelines on how these studies should be conducted and reported. This lack of transparency has led to the misperception that health workforce models operate as ‘black boxes’, without a clear explanation of statistical approaches, assumptions and data sources, and model validity.

The aim of this paper is to conduct a systematic review of recent literature on health workforce projection and forecasting, with the dual purpose of summarizing key findings and proposing comprehensive good practice reporting guidelines. Specifically, we aim to: (1) systematically review and synthesize the existing literature on methods used in health workforce projection models to identify key findings, trends, and methodological approaches; (2) critically assess the methodological quality, strengths, and weaknesses of the existing literature, with a focus on its relevance in answering policy and scenario analysis; (3) develop good practice reporting guidelines that encompass essential elements of study design, data, methodology, and reporting specific to health workforce projection models.

## Methods

This systematic review adhered to a pre-established protocol and was registered with PROSPERO (registration number CRD 42023407858).

### Systematic review of peer-reviewed papers

We conducted a systematic review of recent academic papers focused on health workforce projection and forecasting models published since 2010.

#### Database

In August 2023, we systematically searched electronic databases, including Ovid Medline, EMBASE, and Scopus. The search encompassed articles published from January 1, 2010, to June 2023. The time frame was limited to recent studies to focus on the most relevant and up-to-date information. Additionally, the bibliographies of included articles were screened to identify additional studies meeting the inclusion criteria.

#### Search strategy

Our search strategy involved the utilization of relevant keywords and Medical Subject Headings (MeSH) terms. The keywords for health workforce were ‘health workforce’, ‘health professionals’, and ‘human resources’. The keywords for projection and forecasting models were ‘planning’, ‘model’, ‘forecast’, and ‘projection’.

#### Inclusion and exclusion criteria

Population, Intervention, Comparison, Outcome, Study Design (PICOS) inclusion and exclusion criteria are outlined in Table 1.

**Table 1:** Inclusion and exclusion criteria

	Included	Excluded
Population	Any population, no limitation on country. Focus on workforce planning and projection in the health sector	Studies focusing on workforce planning and projection outside the health and aged care sector
Intervention	A quantitative model for estimating current and future health workforce requirement Estimate both the demand for and supply of workforce and presented the workforce requirements	No forecast/prediction beyond current situation Only either demand for workforce, or supply of workforce exclusively
Comparison	Studies involving quantitative analysis and models that forecast or project future health workforce needs	Conceptual discussion models focusing solely on past and current event
Outcomes	This study aims to review health workforce modelling studies that estimate workforce numbers demand and supply using quantitative methodology	Studies that do not estimate workforce requirements. Studies solely focus on supply or demand side without considering both aspects
Types of studies	Peer-reviewed articles published in academic journals since 2010. Publish in English. All types of quantitative study designs. Full-text articles available for review	Conference abstracts, editorials, commentaries, narrative, qualitative only studies, opinions, clinical case reports, review studies,

#### Data extraction

A standardized data extraction form was developed to systematically extract relevant information from the included studies. Key data elements included study characteristics (e.g. author, publication year, study design), methodology, data sources, variables utilized in the models, and the models’ application in addressing specific health policy issues.

The process of developing the reporting guideline engaged a panel of experts with diverse backgrounds in health workforce planning. This included specialists in health workforce modelling (IC, JTL, MT, JL), health workforce data (DM), and health workforce implementation (MC). Furthermore, experts experienced in journal editing (JTL) and decision-making (MC, FX, TS) participated in the discussions. This collaborative approach leveraged their collective expertise to ensure the academic rigour of the reporting guideline and its relevance in policymaking.

## Results

**Fig. 1:**
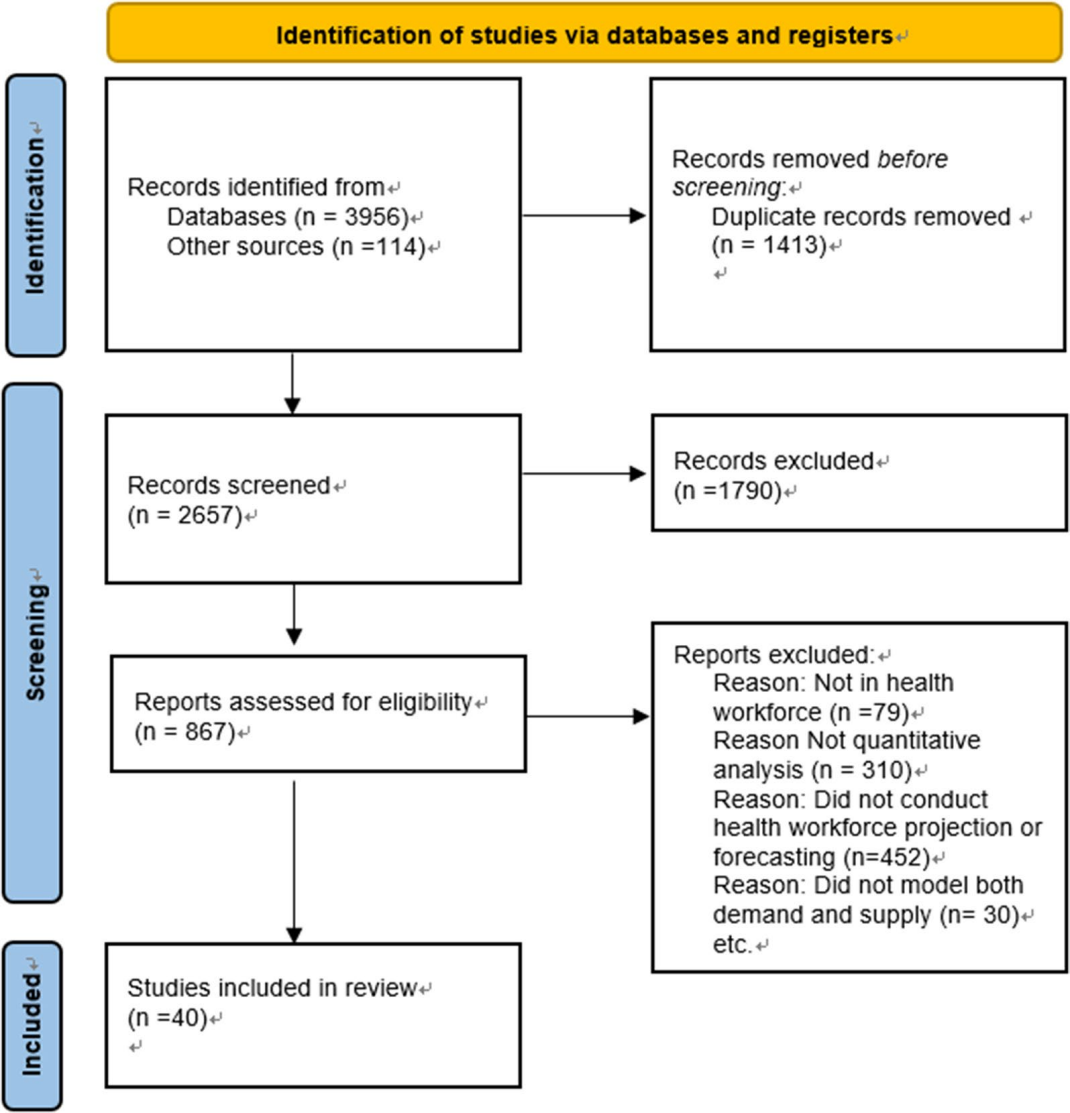
PRISMA flowchart for the systematic review

We retrieved 4070 citations from bibliographic databases, supplemented by 73 citations from other sources. After removing duplicates, we screened 2657 unique citations by title and abstract, resulting in 336 full-text articles for further evaluation. Out of these, 292 studies were excluded based on predefined criteria, and ultimately, 40 studies met the final inclusion criteria. The PRISMA flowchart of the study identification process is presented in Fig. 1.

### Characteristics of included articles

The systematic review covered 40 articles from various countries, including 39 single-country studies and one multiple-country study. Over 70 percent of studies concentrated in high-income countries. Single-country studies represented a range of countries, including Australia (*n* = 3) [14–16], Canada (*n* = 6) [17–22], Germany (*n* = 1) [23], Ghana (*n* = 2) [24, 25], Guinea (*n* = 1) [26], Ireland (*n* = 1) [27], Jamaica (*n* = 1) [28], Japan (*n* = 1) [29], Kazakhstan (*n* = 1) [30], Korea (*n* = 1) [31], Lesotho (*n* = 1) [32], Malawi (*n* = 1) [33], New Zealand (*n* = 2) [34, 35], Portugal (*n* = 1) [36], Saudi Arabia (*n* = 1) [37], Serbia (*n* = 1) [38], Singapore (*n* = 2) [39, 40], Spain (*n* = 1) [41], Thailand (*n* = 3) [42–44], the United Kingdom (*n* = 2) [45, 46], and the United States (*n* = 6) [47–52]. The multiple-country study focused on 32 OECD countries [53].

Among the included studies, 30 focused on national analysis, and only ten focused on sub-national analyses, examining specific regions or areas within a country. The most considered health professionals in the models were physicians and general practitioners (GPs) (*n* = 17), nurses (*n* = 12), and dental professionals (*n* = 4). The mean and median projected duration in the models reviewed were 15.7 and 15.0 years, respectively. A detailed description of each article can be found in Additional file 1: Table S1.

### Components of the health workforce projection model

**Fig. 2:**
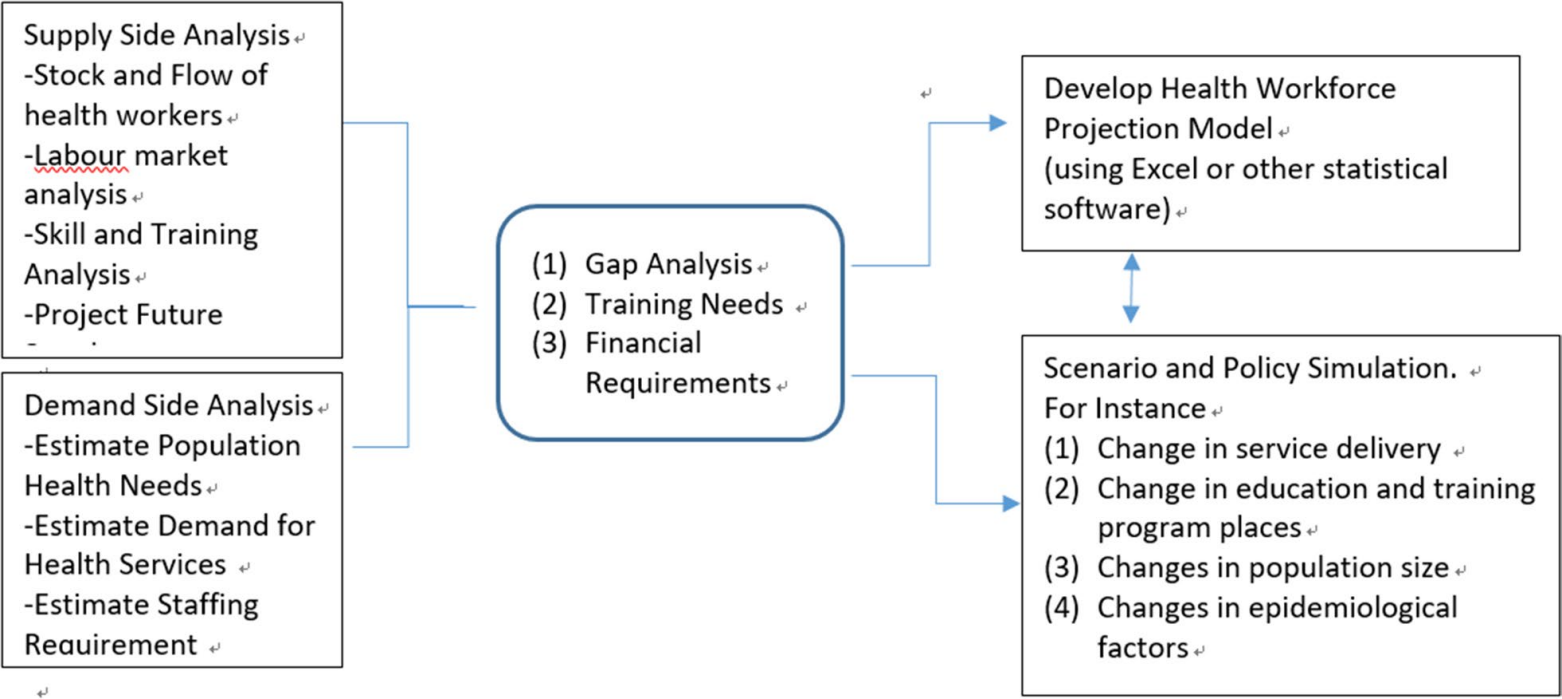
Components of health workforce planning model

Nearly all the included studies describe the overarching analytical framework for the health workforce planning model. Figure 2 summarizes the analytical framework, consisting of three components: demand-side analysis, supply-side analysis, and gap analysis (including training and financial resources needs). It is worth noting that very few studies also incorporate budgetary analysis (*n* = 5) into the gap analysis to examine the financial feasibility of closing the supply–demand gap. The primary tool employed in building these models is Excel spreadsheets.

### Supply and demand analytical framework

We identified eight common supply and demand projection frameworks: population-to-provider ratios (*n* = 7), utilization-based (*n* = 10), needs-based (*n* = 25), skill-mixed (*n* = 5), stock-and-flow (*n* = 40), agent-based simulation (*n* = 3), system dynamic (*n* = 7) and budgetary model (*n* = 5). Studies often combine these models to project the demand and supply of the health workforce. We further summarize the relative strengths and limitations of each model in Additional file 1: Table S2.

It is worth noting that included studies often combine these models for demand, supply, and gap analysis. Additionally, we identified seven statistical approaches commonly used for estimation: arithmetic calculation, optimization, time series analysis, econometric regression, microsimulation, cohort-based simulation, and feedback causal loops (see Table 2 for model and approach details).

**Table 2:** Statistical approaches within different health workforce projection models

	Arithmetic calculation	Optimization	Time-series analysis	Econometrics regression modelling	Microsimulation	Cohort-based simulation model	Feedback and causal loop analysis
Population-to-provider ratio model	v		v				
Utilization model			v	v			
Needs-based model			v	v			
Skill-mixed model		v	v				
Stock-and-flow model	v		v				
Agent-based simulation model					v		
System dsynamic model			v	v		v	v
Budgetary model	v			v			

### Demand-side analysis

The Provider-to-Population model estimates health workforce requirements as a proportion of the population. A common statistical approach used for this model is arithmetic calculation. For example, Lupu et al. [47] adopted this model to calculate the workforce requirements for hospice and palliative care as a fraction of the 65+ population in the US. More sophisticated approaches like time series analysis have also been applied. Milicevic et al. [38] estimated a polynomial least squares model to project the population requiring public health specialists in Serbia. The provider-to-population model is simple and easy to use. However, it disregards skills, distribution, and specific healthcare needs. Another limitation arises from the challenge of determining the “ideal” ratio of providers to the population, which is subjective and influenced by healthcare needs, geography, and cultural context.

Utilization (or demand-based) models can overcome the above limitation by estimating health service needs based on historical utilization. Since historical data are used, the most common statistical approaches used for this model are econometrics and time series analysis. For instance, Jager et al. [23] projected dental services using least-square estimation, accounting for factors like age, gender, and socioeconomic status. This straightforward approach enables policymakers to incorporate demographic factors into the demand projection. However, it is important to note that utilization-based models also have limitations. They heavily rely on past or current utilization patterns, which may overlook unmet needs or instances of overutilization within the population. This can be mitigated by adjusting for unmet needs, as done by Landry et al. [52].

To address over-utilization in the demand projection, an alternative solution to model demand is to use needs instead of utilization. The schema of the conceptual framework of the needs-based model is illustrated in Additional file 1: Fig. S1. The needs-based model, found in most included studies, explicitly considers population health needs, particularly epidemiological factors like health conditions and health status. For example, Tomblin Murphy et al. [53] estimated the needs for general practitioners to deliver primary care in Canada. Time series analysis is also used for predicting demographic and epidemiological trends in this model. An example is Al-Senani et al. [37], who predicted stroke incidence using epidemiological modelling to determine workforce requirements. Since the model relies heavily on disease prevalence data, these studies focus more on a single health condition than a broader range of health conditions. Moreover, the model also does not account for changes in the delivery of health care due to the missing utilization data.

The skill-mix model, applied in several studies, uses optimization models to optimize the allocation of healthcare professionals, considering skills, capacity, scope of practice, and capability. An advantage of this model is the ability to account for substitution between providers of the same levels (*horizontal substitutions*) and between providers of different levels (*vertical substitutions*). For example, Gallagher et al. [45, 46] used an optimization model to identify the optimal healthcare workforce mix for dental services in the UK. Both papers used linear programming, which assumes workforce requirements as a linear function of dental care demand estimates, staff competency, cost, and volume of activity. Then, an optimal staff mix can be numerically computed. Despite its uses in decision-making, the skill mix model relies heavily on skill data, which is sometimes unavailable. Incomplete or inaccurate data can affect the accuracy of the model's reflection of the actual distribution of skills within the healthcare workforce. Additionally, the model may not sufficiently consider factors such as patient preferences or cultural aspects that can influence the distribution of healthcare providers. Lastly, the skill-mix model can be difficult for non-mathematicians to use since it builds on the optimization process, which can be computationally complex [54].

### Supply-side analysis

Supply-side analysis typically begins with a stock-and-flow model, which examines workforce variables such as size, education, migration, and attrition. The input variables can be estimated using either simple mathematical formulas or more advanced time series analysis. The model also estimates clinical Full-Time Equivalents (FTE) based on participation rates and direct clinical time. The stock-and-flow model provides a simplified yet effective framework for analysing the supply side of the health workforce. However, it may not capture the heterogeneity in the workforce (e.g. age) and its interaction with the system (e.g. changes in minimum wages).

Agent-Based Simulation (ABS) and System Dynamic (SD) models can address these limitations. The ABS adopts the microsimulations approach to simulate the behaviour and interactions of individual agents, such as healthcare workers, within a given system, considering their interactions with the broader policy and social-environmental context. For example, Lopes [36] built an ABS model using Portuguese administrative data to predict the healthcare workers’ decision to work, providing a more detailed prediction of the anticipated supply. They built the model in AnyLogic, a free software tool for simulation. Another free software that can be used for microsimulation is R. The software has been used by the US. Health Resources and Services Administration to produce their health workforce model and dashboard [55].

Unlike ABS models, SD models operate at the cohort or group levels, accounting for feedback loops and delays. Thus, it enables the interconnectivity of different model components. For example, Dill et al. [49] utilized this model to estimate the supply of physicians in the US. The model builds on the stock-and-flow model but allows it to respond to demand changes and vice versus. For example, when supply exceeds demand, immigration may slow down. The model is built in free simulation software, Vensim. Another SD software is Stella, which is a web-based modelling tool. However, the software is free for only limited functions. Although these two models are helpful in predicting changes in the labour supply because of system changes, both methods are time-consuming and resource-intensive, requiring detailed unit-level data and specific software knowledge.

### Gap analysis and budgetary analysis

Gap analysis is a common element in all studies, presenting demand–supply gaps in absolute numbers and sometimes relative gaps. Additionally, some studies incorporate budgetary analysis, as seen in Asamani et al. [32], which assesses the financial implications of filling supply–demand gaps and evaluates affordability within public health budgets.

### Data prerequisites, variables, and sources

Most included studies described data and variables for their models. Table 3 summarizes data prerequisites for various models.

**Table 3:** Data prerequisites for various health workforce projection models

Health workforce projection model	Provider-to-population model	Utilization model	Needs-based model	Skill-based model	Stock-and-flow model	Agent-based simulation model	System dynamic model	Budgetary analysis
Demographic measures	Basic population counts and projections	Age/gender disaggregated population counts and projections	Age/gender disaggregated population counts and projections					
Measures of epidemiology			Prevalence, incidence of health conditions and risk factors. General health status					
Health service utilization measures		Rate of health service utilization by age/gender	Recommended health service interventions for the health condition					
Health service delivery measures				Expert opinion for optimal skill-mix for service delivery				
Work standard and productivity measures		Professional regulations. Standard workload	Professional regulations. Standard workload	Professional regulations. Standard workload				
Supply measures					Current stock of health workforce. Education and training pipeline. Participation rate and activity level. Mobility data. Immigration Data			
Behavioural roles and microeconomics foundations						Individual demographic, socioeconomic status, behavioural data		
Feedback loops							Feedback loop on how actors' response to difference scenarios	
Financial and budgetary measures								Salary, Cost of education and training. Available government health budget

Demand-side analyses increase data complexity from the Provider-to-Population Model to the Utilization and Needs-Based Models. The Provider-to-Population Model requires basic demographic data, while the Utilization Model adds age and gender-based health service utilization rates. The Needs-Based Model extends this to epidemiological data, health interventions, and service delivery recommendations. Skill-mix models need comprehensive data for optimizing the workforce. Except for the Provider-to-Population Model, all demand-side models require productivity and workload data to estimate health workforce needs based on service requirements.

Supply-side models have specific data needs. The Stock and Flow Model needs current health workforce, education, mobility, and migration data. Agent-Based Simulation Models use individual behaviour data. System Dynamic Models focus on feedback loops, while Budgetary Analysis requires staff costs, education expenses, intervention costs, and government health budget data.

Our findings indicate a prevalent use of assumptions in the models, with many parameters derived from prior literature or expert opinions. Notably, sub-national analyses often suffer from a lack of local-specific data, while even national analyses often have challenges with data availability and accessibility. For instance, in a needs-based analysis of physicians, midwives, and nurses in 32 OECD countries, Tomblin-Murphy et al. [53] found that only 35% of the data elements required to implement the projection model were available. The most common available variables were population size, general health status of the population, head count of current supply, new graduates per year, health workforce participation rate, salary. Other variables have minimal availability.

### Methods for model validation and uncertainty

We found limited number of the studies, incorporating model validation and uncertainty analysis (see Additional file 1: Table S4). These analyses comprise three key aspects: external model validation of the model structure (*n* = 4), internal validation of prediction accuracy (*n* = 8), and sensitivity analysis to assess parameter uncertainty (*n* = 13).

External model validation is used to confirm the accuracy of the high-level model structure in the system dynamic model and evaluate the behavioural assumptions in the agent-based simulation models. To achieve this, researchers often engage in panel discussions with groups of experts in healthcare services, systems research, and government agencies. The aim is to reach a consensus that the model structure accurately reflects real-world situations [43].

On the other hand, internal validation primarily focuses on demonstrating that the model's predictions closely align with actual values. All included papers conduct internal validation by comparing model estimates with historical data. The statistical analysis method used in this validation process often involves calculating the normalized measure of error or root-mean-square percentage error (RMPSE). A commonly accepted rule of thumb is that an RMPSE of less than 10% is considered reasonable [40, 56].

Regarding uncertainty stemming from parameters in the model, two types of sensitivity analysis are normally conducted in the included studies: probabilistic and deterministic analysis. The first approach utilized in Lopes et al. [36], is the Markov Chain Monte Carlo (MCMC) analysis. This involves generating replicated samples of parameters and running simulations multiple times to estimate the probability distribution of model values, allowing for the generation of confidence intervals in the estimates. The second approach commonly used to address parameter uncertainty involves comparing estimates derived from high and low parameter values. For example, Anash et al. [56] assessed model uncertainty by comparing optimistic and pessimistic scenarios.

### Policy and scenario analysis

Most studies (32 out of 40) conducted policy and scenario analysis, as detailed in Additional file 1: Table S4. This analysis changes one or some parameters in the model and identifies changes in the outputs compared to a status quo. The common parameter changes include changes in population size (*n* = 7), epidemiological characteristics (*n* = 5), alternative care delivery models (*n* = 11), levels of health insurance use (*n* = 6), intervention affects labour participation of health workforce (*n* = 12), increases in education and training placements (*n* = 14), the number of overseas immigrants (*n* = 3), and changes in working hours and productivity levels (*n* = 14).

Most studies model multiple scenarios simultaneously rather than a single intervention alone. For example, Tomblin-Murphy [17] models the following policies in isolation, as well as the combined effect of the following policies: increased education places, reduced attrition in education programmes, improved retention in workforce, reduced absenteeism, reduced in-migration of foreign-trained workforce, increase productivity level.

### Recommended health workforce projection model reporting guideline

One of the key findings of our study is the lack of standardization in reporting methods within existing studies. In response, we have proposed a “Good Practice Reporting Guideline for Health Workforce Models” (Table 4). The “Good Practice Guideline for Health Workforce Reporting” comprises 30 items that provide a structured framework for clear and comprehensive reporting in health workforce projection studies. These items cover critical aspects, including study identification, population characteristics, geographical considerations, analytical methods, results presentation, demand–supply gap analysis, and financial assessments. Furthermore, the guideline emphasizes the significance of addressing uncertainty, assumptions, and model validation.

**Table 4:** Good practice reporting guideline for health workforce projection models

Section	Item no.	Guidance for reporting
Title		
Title	1	Identify the study as health workforce planning and specify the health professionals being estimated
Abstract		
Abstract	2	Provide a structured summary that highlights context, key methods, results, and alternative analyses
Introduction		
Background and objectives	3	Give the context for the study, the study aims and objectives, and its practical relevance for decision-making in policy or practice
Methods		
Study population	4	Describe characteristics of the study population
Geographical area	5	Describe the geographical area of the study Report the level of geographical area that the HWP models is applied to
Health condition	6	Describe whether the study focuses on a specific health condition
Health professionals	7	Describe which health professional is the focus of the study
Time horizon	8	Describe the time horizon of the project and forecasting model
Scope of activities	9	Specify whether the study exclusively focus on healthcare or if it also includes age care, disability care, and community care
Overall analytical framework	10	Discuss the analytical framework used in the study
Study parameters	11	Describe specific parameters and variables used in the model, and explain how they were estimated
Models and statistical approaches for demand-side analysis	12	Explain how demand for health workforce was estimated
Models and statistical approaches for supply-side analysis	13	Explain how the supply of health workforce was estimated
Methods and statistical approaches for gap analysis	14	Describe the methodology for calculating the gap between supply and demand, and how sufficiency of health professionals is presented
Methods and statistical approaches for budgetary analysis	15	Explain the methodology for the financial and budgetary assessment
Characterizing uncertainty	16	Discuss how uncertainty was quantified and addressed. Explain how estimates for parameters were determined
Assumptions of the model	17	List and justify key assumptions made in the projection and forecasting model
Methods for validation of the model	18	Explain how the model's accuracy and reliability were validated. Describe how parameters in scenario analysis were derived
Data source	19	Provide a comprehensive list of all data sources used, with details on quality and relevance
Results		
Summary of projection results	20	Provide supply and demand projections for each healthcare profession
Projection for different geographical areas (if applicable)	21	Include projections for various regions or healthcare facilities if applicable
Identify supply–demand gap in health professionals	22	Present a clear assessment of workforce shortages or surpluses over time
Identify geographical distribution (if applicable)	23	Present a clear assessment of the geographical distributional analysis to determine relatively underserved and overserved population
Effects of policies and scenarios (if applicable)	24	Discuss the health workforce requirement impact of different policy scenarios on workforce planning. Possibly the financial and budgetary impact
Effects of uncertainty and sensitivity analysis (if applicable)	25	Analyse the effects of uncertainty and sensitivity on projection outcomes
Model validation (if applicable)	26	Highlight the validity and robustness of the model and its sensitivity to changes in assumptions
Discussion		
Key findings, limitation,	27	Discuss implications of findings for health workforce projection and potential challenges or constraints
Consideration of policies related to planning, education, training, recruitment, and retention	28	Offer policy recommendations to address workforce gaps and improve healthcare delivery
Other relevant information		
Source of funding	29	Disclose all sources of funding for the study
Conflict of interests	30	Provide information about any potential conflicts of interest among the study’s authors or contributors

## Discussion

### Principal findings

Our study provides a comprehensive review of recent advancements in the methods for health workforce projection and forecasting models. In our study, we have identified eight different model types covering supply, demand, and budgetary components of the health workforce projection model. It is important to acknowledge that no model can be considered flawless, as each one operates under its unique set of assumptions, strengths, and limitations. Health workforce modelers frequently used these eight models in combination. Our findings suggest that best practices incorporate considerations of both the drivers of supply and demand for healthcare services, in addition to epidemiological needs, shifts in productivity, skill mix dynamics, policy alternatives, and budgetary requirements.

Regarding statistical techniques, we have identified seven common statistical approaches used in health workforce projection models, and the use of these approaches is highly contingent on data availability. These techniques are commonly used and have their roots in the fields of health economics, operational, epidemiological, and health service research. It is also noteworthy that workforce projection often relies on imperfect data with limited granularity at the local level.

Furthermore, it is important to acknowledge the diversity of health workforce projection models and statistical approaches in the literature, where existing studies often lack uniformity in reporting their methodologies. While some health workforce modelers consider health workforce modelling a blend of science and art [57, 58], our best-practice reporting guidelines aim to provide a comprehensive framework for future research in this field. To tackle this diversity, our recommended guidelines are designed to accommodate various model types, emerging methodologies, and the growing utilization of advanced statistical techniques to address uncertainties and the urgent need for model validation.

### Research recommendations

Based on these findings, several research recommendations emerge. First, as the size of the health workforce continues to grow and health workforce projection and forecasting models become increasingly complex, there is a strong need to adopt standardized reporting criteria for health workforce projection models. A common reporting framework can be a catalyst for transparency and model quality.

Secondly, our findings reveal a growing trend in aligning health workforce projection models with underlying health needs and considering the implications of different health service delivery models. It is essential to continue developing methodologies for needs-based, multi-professional, skill-mixed models, particularly considering recent trends such as an aging population, an increased prevalence of chronic diseases, and patients with multiple conditions. As patients increasingly require patient-centred care delivered by a multi-professional team, there is a methodological challenge in effectively modelling team-based care [59, 60]. This shift away from profession-specific approaches in favour of a more team-oriented approach is necessary to address the evolving workforce requirements in healthcare.

Our study revealed a need for more research that addresses the mal-distribution of the health workforce across different geographical areas. To address this issue, it is crucial to develop methodologies for assessing geographical distribution and examining the relative over-service or under-service of health professionals [61]. While financial resources are crucial for health workforce planning implementation, existing literature suggests that many current models neglect budgetary analyses. Among the few studies that include budgetary analysis, none considered the potential cost offsets resulting from improved health outcomes [62]. Explicitly integrating financial and budgetary analysis into health workforce projection models, rather than treating it as an add-on, is highly desirable.

Our study focuses on specific professions like GPs, nurses, and dentists in health workforce planning. This narrow focus on only a few health professionals may be attributed to the lack of data on other professionals, such as allied health and aged care workers. To enhance workforce planning, it is crucial to improve data collection for these occupations. Data collection should also consider contemporary work patterns, where health professionals may work part-time and allocate varying proportions of their time to clinical practice. Additionally, data on the mobility and behaviour of health professionals, including their movement across geographical areas, will significantly enhance the accuracy of simulation models.

Finally, our study revealed that existing research predominantly relies on static system frameworks rather than dynamic analyses of the interconnected ‘systems’ inherent in health workforce planning. Existing models have been largely linear, instead of systems-based, regarding their methodologies [49]. It is imperative for researchers to shift towards a complex system-thinking approach within health workforce projection models, departing from static models. This shift involves comprehending the dynamic and interrelated nature of healthcare systems and considering the potential existence of feedback loops under different scenarios. This holistic perspective is essential for a more comprehensive understanding of workforce planning dynamics.

## Limitations

This paper has a few caveats. Firstly, it focuses on studies that project both health workforce demand and supply while calculating the demand–supply gap. This exclusion applies to research that exclusively addresses either the demand or supply of the health workforce, as well as cross-sectional studies. Secondly, the review only includes studies published after 2010, potentially missing older studies. Nevertheless, this approach was chosen to encompass the representation of recent developments in the field and highlight their significance and innovation.

## Conclusion

Health workforce projection models are complex analyses of various interconnected systems of demand for and supply of health workforce, as well as the resources required to meet the demand–supply gap. Our study underscores the importance of dynamic, multi-professional, and fine-tuned demand, supply, and budget impact analyses, supported by robust health workforce data intelligence. The suggested best-practice reporting guidelines aim to promote transparency in health workforce projection models and provide valuable support to healthcare practitioners and researchers.

### Supplementary Information

**Additional file 1: Table S1.** Characteristics of the included studies. **Table S2.** Strength and limitation of health workforce analytical framework. **Table S3.** Model validation and uncertainty. **Table S4.** Policy and scenario analysis of the included studies. **Figure S1.** Schematic diagram of a needs-based health workforce planning model.

## Data Availability

Not applicable.
