# Supplementary material for: Methods for health workforce projection model: systematic review and recommended good practice reporting guideline
Source: Hum Resour Health. 2024 Apr 17;22:25. doi: 10.1186/s12960-024-00895-z (PMC11025158; doi:10.1186/s12960-024-00895-z)
Supplement: Supplementary file 1 — Additional file 1: Table S1. Characteristics of the included studies. Table S2. Strength and limitation of health workforce analytical framework. Table S3. Model validation and uncertainty. Table S4. Policy and scenario analysis of the included studies. Figure S1. Schematic diagram of a needs-based health workforce planning model. [file 12960_2024_895_MOESM1_ESM.docx]

**Table S1 – Characteristics of the included studies**

| **Study** | **Country** | **National or Subnational** | **Health condition** | **Health profession** | **Time horizon of projection** |
| --- | --- | --- | --- | --- | --- |
|  |  |  |  |  |  |
| Barber et al (2010)^41^ | Spain | National | Not specific | Multiple professionals | 17 years (2008-2025) |
| Gallagher et al (2010)^55^ | UK | National | Dental health | Dental professional | 23 years (2005-2028) |
| Tomblin Murphy et al (2012)^17^ | Canada | National | Not Specific | Nurse | 15 years (2007-2022) |
| Schofield et al (2012)^14^ | Australia | National | Not specific | Radiation oncologists | 5 years (2014-2019) |
| Ishikawa (2013)^67^ | Japan | National | Not specific | (1) clinical physician (2) OB/GYN specialist | 22 years (2008-2030) |
| Gallagher et al (2013)^46^ | UK | Subnational- South Central England | Dental health | Dental professional | 6 years (2007-2013) |
| Jansen et al (2014)^26^ | Guinea | Subnational | Maternal health | Maternal health professional (nurse, midwife, physician, GYN, paediatrician) | 10 years (2014-2024) |
| Vanderby et al (2014)^68^ | Canada | National | Cardiac surgery | Cardiac surgeon | 22 years (2008-2030) |
| Tomblin Murphy et al (2014)^69^ | Jamaica | Subnational- Southeast Region | Not specific | Pharmacist | 15 year (2008-2023) |
| Ansah et al (2015)^39^ | Singapore | National | Eye health | Ophthalmologist | 25 years (2015-2040) |
| Ranta et al (2015)^70^ | New Zealand | National | Neurology | Neurology professional | 12 years (2014-2026) |
| Landry et al (2016) | United States | National | Physical therapy | Physical therapist | 10 years (2010-2020) |
| Jager et al(2016)^23^ | Germany | Subnational-Northern Germany | Dental health | Dentist | 19 years (2011-2030) |
| Laurence et al (2016)^71^ | Australia | Subnational- South and Western Australia | Not specific | GP | 10 years (2003-2013) |
| Tomblin Murphy et al (2016)^58^ | Multiple OECD countries | National | Not specific | Physician, nurse, midwife | 15 years (2015-2030) |
| Oh (2017)^72^ | Korea | National | Urologic condition | Urologist | 18 years (2012-2030) |
| Laurence et al (2018)^73^ | Australia | Subnational- South and Western Australia | Not specific | GP | 20 year (2013-2033) |
| Lupu et al (2018)^47^ | United States | National | Not specific | Hospice and palliative physician | 25 years (2015-2040) |
| Lopes et al (2018)^36^ | Portugal | National | Not specific | Physician | 35 years (2015-2060) |
| Yip et al (2018)^74^ | Canada | National | Cancer | Oncologist | 10 years (2016-2026) |
| Milicevic et al (2018)^38^ | Serbia | National | Not specific | public health specialist | 10 years (2015 to 2025) |
| Pagaiya et al (2019)^75^ | Thailand | National | Not specific | Multiple professionals | 10 years (2016 to 2026) |
| Ahern et al (2019) | Ireland | National | Dental health | Dentist | 23 years (2017-2050) |
| Ansah et al (2019) | Singapore | National | Eye health | Multiple professionals | 25 years (2015-2040) |
| Al-Senani et al (2019) | Saudi Arabia | National | Stroke | Multiple professionals | 10 years |
| Mackenzie et al (2019 | Canada | Subnational- Nova Scotia | Mental health | Mental health nurses/ physicians/ psychologists/ social worker | 14 years (2018-2032) |
| Thongsukdee et al (2019) | Thailand | National | Not specific | Physician | 26 years (2014-2040) |
| Taghavi et al (2020) | Canada | Subnational- Nova Scotia | Palliative Care | Multiple professionals (nurse, physician, social worker) related to palliative care | 19 years (2019-2038) |
| Russo et al (2020) | United States | National | Hepatology care | Multiple hepatology professionals | 15 years (2018-2033) |
| Leerapan et al (2021) | Thailand | National | Not specific | Multiple professionals | 19 years (2018-2037) |
| Asamani et al (2021a) | Ghana | National | Maternal health | Multiple professions related to maternal health | 10 years (2020-2030) |
| Asamani et al (2021b) | Ghana | National | No | Multiple professions | 15 years (2020-2035) |
| Chen et al (2021) | United States | National | COVID-19 | COVID related professionals (ICU physicians, nurses, respiratory therapist, public health doctors) | 6 months |
| Dill et al (2021) | United States | Subnational- Cleveland and Albuquerque | Not specific | Physicians (including both primary care and specialty care physicians) | 20 years (2015-2035) |
| Koichubekov et al (2021) | Kazakhstan | National | Not specific | General practitioner | 12 years (2018-2030) |
| Berman et al (2022) | Malawi | National | No | Multiple professionals | 4 years (2018-2022) |
| Dass et al (2022) | Canada | Subnational- Ontario | Long-term care | Personal support worker/ Nurse | 15 years (2020-2035) |
| Asamani et al (2022) | Lesotho | National | Not specific | Multiple professionals | 10 years (2020-2030) |
| Correll et al (2022) | United States | National | Rheumatology disease | Multiple professions | 15 years (2015-2030) |
| Dunn et al (2022) | New Zealand | National | Cancer | Radiation oncologists | 10 years (2021-2031) |

**Table S2 – Strength and limitation of health workforce analytical framework**

|  | **Model** | **Strength** | **Limitation** |
| --- | --- | --- | --- |
| Demand analysis | Population-to-Provider model | - Simplicity - Ease of use | - Oversimplified - Disregard skills and productivity |
|  | Utilization-based model | - Consider demographic changes (e.g., population ageing) | - Data-dependent - Disregard unmet needs |
|  | Need-based model | - Consider changes in health needs/disease prevalence | - Data-dependent - Do not account for team-based care - Disregard patient preferences or societal benefits |
|  | Skill-mixed model | - Consider team-based care (horizontal and vertical substitution) | - Data-dependent - Disregard patient preferences |
| Supply analysis | Stock-and-flow model | - Simplicity - Ease of use | - Oversimplified |
|  | Agent-based simulation model | - Capture dynamic and complexity in the system - Simulate impacts of future incentives | - Resource-intensive and time consuming - Unit-data dependent |
|  | System dynamic model | - Capture dynamic and complexity in the system - Simulate impacts of future incentives - Consider feedback loop | - Resource-intensive and time consuming - Unit-data dependent |

**Table S3 – Model validation and uncertainty**

| Study | **Model Validation and Uncertainty** | | |
| --- | --- | --- | --- |
|  | **Internal Model Validation** | **External Model Validation** | **Sensitivity analysis of parameter uncertainty** |
| Barber et al (2010) | No | No | No |
| Gallagher et al (2010) | No | No | Yes |
| Tomblin Murphy et al (2012) | No | No | No |
| Schofield et al (2012) | No | No | No |
| Ishikawa (2013) | Yes | No | Yes |
| Gallagher et al (2013) | No | No | No |
| Jansen et al (2014) | No | No | No |
| Vanderby et al (2014) | No | No | No |
| Tomblin Murphy et al (2014) | No | No | No |
| Ansah et al (2015) | Yes | Yes | Yes |
| Ranta (2015) | No | No | No |
| Landry (2016) | No | No | No |
| van den Berg (2016) | No | No | Yes |
| Laurenece et al (2016) | No | No | No |
| Tomblin Murphy (2016) | No | No | Yes |
| Oh (2017) | No | No | No |
| Laurence et al (2018) | No | No | No |
| Lupu et al (2018) | No | No | No |
| Lopes et al (2018) | No | No | Yes |
| Yip et al (2018) | No | No | No |
| Milicevic (2018) | No | No | No |
| Pagaiya et al (2019) | No | No | Yes |
| Ahern et al (2019) | no | no | no |
| Ansah et al (2019) | Yes | Yes | Yes |
| Al-Senani et al (2019) | No | No | No |
| Mackenzie et al (2019 | No | No | No |
| Thongsukdee et al (2019) | No | No | Yes |
| Taghavi et al (2020) | Yes | No | No |
| Russo (2020) | No | No | Yes |
| Leerapan et al (2021) | No | Yes | No |
| Asamani et al (2021a) | Yes | No | No |
| Asamani et al (2021b) | No | No | Yes |
| Chen et al (2021) | No | No | No |
| Dill et al (2021) | Yes | Yes | No |
| Koichubekov et al (2021) | Yes | No | No |
| Berman et al (2022) | No | No | No |
| Dass et al (2022) | No | No | No |
| Asamani et al (2022) | No | No | No |
| Correll et al (2022) | No | No | Yes |
| Dunn et al (2022) | Yes | No | Yes |

**Table S4 - Policy and scenario analysis of the included studies**

|  | **Policy and scenario analysis** | | | | | | | | | | |
| --- | --- | --- | --- | --- | --- | --- | --- | --- | --- | --- | --- |
|  | **Changes in population growth** | **Changes in epidemiological characteristics** | **Alternative care deliver model** | **Health insurance coverage** | **Changes in level of health service use** | **Intervention affects participation, attrition, or retention of health workforce** | **Changes in training places** | **Changes in Immigration policy** | **Change in retirement age** | **Changes in government budget** | **Change in workload and productivity level** |
| Barber et al (2010) | Yes | No | No | No | No | No | No | No | No | No | No |
| Gallagher et al (2010) | No | No | Yes | No | No | No | No | No | No | No | No |
| Tomblin Murphy et al (2012) | No | No | No | No | No | Yes | Yes | Yes | No | No | No |
| Schofield et al (2012) | No | Yes | Yes | No | Yes | No | No | No | No | No | Yes |
| Ishikawa (2013) | No | No | No | No | No | No | Yes | No | No | No | No |
| Gallagher et al (2013) | No | No | Yes | No | No | No | No | No | No | No | No |
| Jansen et al (2014) | No | No | No | No | No | Yes | Yes | No | No | No | No |
| Vanderby et al (2014) | Yes | No | No | No | No | No | No | No | No | No | Yes |
| Tomblin Murphy et al (2014) | No | No | No | No | No | Yes | Yes | No | No | No | Yes |
| Ansah et al (2015) | No | No | Yes | No | No | No | No | No | No | No | Yes |
| Ranta (2015) | No | No | No | No | No | No | Yes | No | No | No | Yes |
| Landry (2016) | Yes | No | No | No | No | Yes | Yes | No | No | No | No |
| van den Berg (2016) | No | Yes | No | No | No | No | Yes | No | Yes | No | Yes |
| Laurenece et al (2016) | Yes | No | No | No | No | Yes | No | No | No | No | Yes |
| Tomblin Murphy (2016) | Yes | Yes | Yes | No | Yes | Yes | Yes | No | No | No | Yes |
| Oh (2017) | No | No | No | No | No | No | No | No | No | No | Yes |
| Laurence et al (2018) | No | Yes | No | No | Yes | No | No | No | No | No | No |
| Lupu et al (2018) | No | No | No | No | No | No | Yes | No | No | No | No |
| Lopes et al (2018) | No | No | No | No | No | No | Yes | No | No | No | No |
| Yip et al (2018) | No | No | No | No | No | Yes | Yes | No | No | No | No |
| Milicevic (2018) | Yes | No | No | No | No | Yes | No | No | No | No | No |
| Pagaiya et al (2019) | No | No | No | No | No | No | No | No | No | No | No |
| Ahern et al (2019) | No | No | No | No | No | No | No | No | No | No | Yes |
| Ansah et al (2019) | No | No | Yes | Yes | Yes | No | No | No | No | No | Yes |
| Al-Senani et al (2019) | No | No | No | No | No | No | No | No | No | No | No |
| Mackenzie et al (2019 | No | No | Yes | No | No | No | No | No | No | No | No |
| Thongsukdee et al (2019) | No | No | No | No | No | Yes | Yes | No | No | No | No |
| Taghavi et al (2020) | No | No | No | No | No | No | No | No | No | No | No |
| Russo (2020) | Yes | Yes | No | No | No | Yes | Yes | Yes | No | No | No |
| Leerapan et al (2021) | No | No | Yes | No | No | No | No | No | No | Yes | No |
| Asamani et al (2021a) | No | No | Yes | No | No | No | No | No | No | No | Yes |
| Asamani et al (2021b) | No | No | Yes | No | No | No | No | No | No | No | Yes |
| Chen et al (2021) | No | No | No | No | No | No | No | No | No | No | No |
| Dill et al (2021) | No | No | No | No | No | Yes | Yes | Yes | No | No | No |
| Koichubekov et al (2021) | No | No | No | No | No | No | No | No | No | No | No |
| Berman et al (2022) | No | No | No | No | No | No | No | No | No | No | No |
| Dass et al (2022) | No | No | Yes | No | No | Yes | No | No | No | No | No |
| Asamani et al (2022) | No | No | No | No | No | No | No | No | No | No | No |
| Correll et al (2022) | No | No | No | No | No | No | No | No | No | No | No |
| Dunn et al (2022) | No | No | No | No | No | No | No | No | No | No | Yes |

***
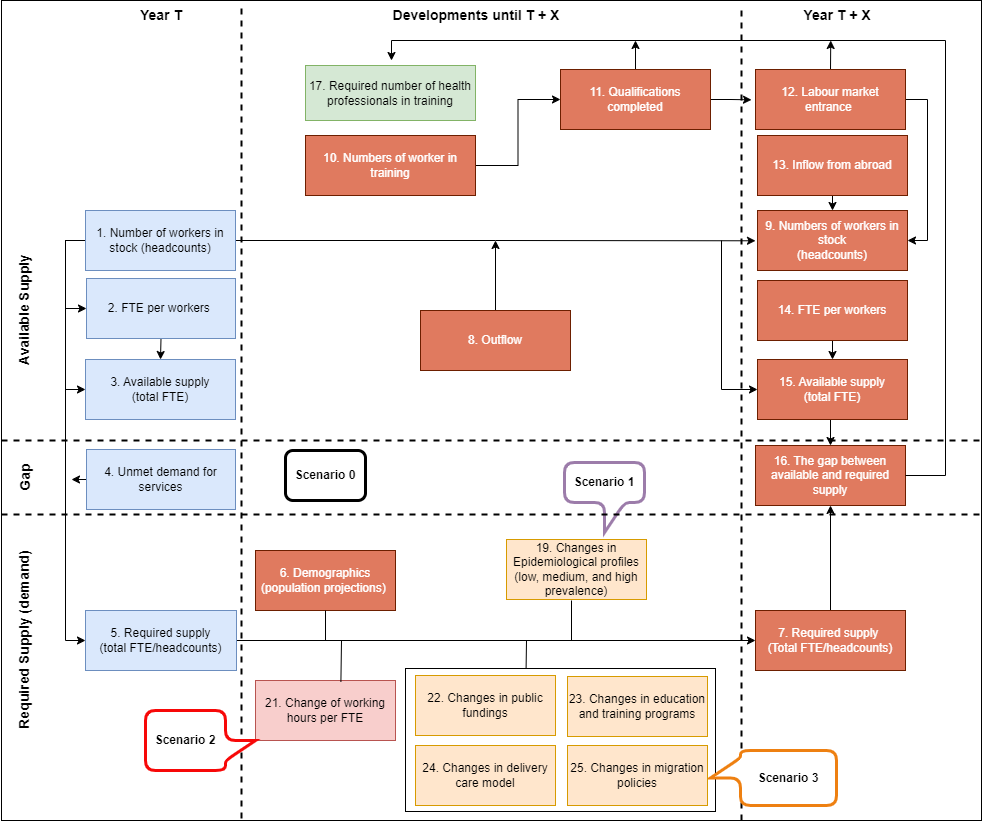
Figure S1 - Schematic diagram of a needs-based health workforce planning model***

We provide a visual representation of the needs-based workforce planning model included our review. This model can be divided into three key stages, depicted as columns in the Figure. The elements on the left-hand side pertain to the baseline year, denoted as year T, while those on the right-hand side focus on the target year (year T+X), a point in the future we aim to project to. The overarching goal of our model is to achieve a balance between the supply and demand for health and care workers in the target year.

The calculation of workforce needs is influenced by various factors, including population requirements, epidemiological profile, health service demands, unmet needs, service delivery models, and productivity (nodes 4, 5, 6, 7, 19, 21, 24). The key consideration in the needs-based model is in node 19 where demand for health workforce is primarily driven by epidemiological profiles.

Meanwhile, the consideration of worker supply includes the existing workforce, latent workforce potential, education and training pipelines, graduates, entrances and exits, domestic inflows and outflows, as well as overseas immigration patterns (nodes 1, 2, 3, 8, 9, 10, 11, 12, 13, 14, 15).

 The balance or gap between demand and supply of workers (node 16) directly impacts the necessary workforce numbers (node 17). Our model is flexible and can simulate changes in vital demographic, epidemiological, and policy variables that affect workforce demand and supply. These variables may include adjustments in demographic factors such as population growth rates (node 6), public funding availability (node 22), education and training programs (node 23), productivity measures such as work standards (node 21), service delivery models (node 24), and migration levels (node 35).
